# Experiences of Home‐Based Pulmonary Rehabilitation With mHealth and Centre‐Based Pulmonary Rehabilitation in People With Chronic Obstructive Pulmonary Disease: A Qualitative Study

**DOI:** 10.1111/hex.70181

**Published:** 2025-02-28

**Authors:** Hannah Rutherford, Marita Dale, Sally Wootton, Rashmi Pithavadian, Sarah Dennis, Sarah Brown, Jennifer A. Alison, Andrew S. L. Chan, Ian A. Yang, Zoe McKeough

**Affiliations:** ^1^ Sydney School of Health Sciences, Faculty of Medicine and Health University of Sydney Sydney New South Wales Australia; ^2^ Strategy, Innovation and Digital Health South Eastern Sydney Local Health District Sydney New South Wales Australia; ^3^ Chronic Disease Community Rehabilitation Service Northern Sydney Local Health District North Ryde New South Wales Australia; ^4^ School of Health Sciences Western Sydney University Campbelltown New South Wales Australia; ^5^ South West Sydney Allied Health Research Collaboration South West Sydney Local Health District Liverpool New South Wales Australia; ^6^ Ingham Institute for Applied Medical Research Liverpool New South Wales Australia; ^7^ Department of Physiotherapy Royal North Shore Hospital St Leonards New South Wales Australia; ^8^ Allied Health, Sydney Local Health District New South Wales Australia; ^9^ Department of Respiratory and Sleep Medicine Royal North Shore Hospital St Leonards New South Wales Australia; ^10^ Northern Clinical School, Faculty of Medicine and Health The University of Sydney Sydney New South Wales Australia; ^11^ The Prince Charles Hospital and Faculty of Medicine The University of Queensland Brisbane Australia

**Keywords:** COPD, mHealth, pulmonary rehabilitation, qualitative study

## Abstract

**Background:**

Mobile health (mHealth) provides innovative solutions to improve access to pulmonary rehabilitation (PR). This study aimed to explore the experiences of people with chronic obstructive pulmonary disease (COPD) who undertook either an 8‐week home‐based PR with a mHealth application (app) (m‐PR) or centre‐based PR (CB‐PR).

**Methods:**

Convenience then purposive sampling was used to recruit participants enrolled in a randomised controlled trial after completion or withdrawal from either m‐PR or CB‐PR. Participants undertook individual, semi‐structured interviews. Interview transcripts were inductively coded and thematically analysed using a critical realist approach.

**Results:**

Thirteen m‐PR and 12 CB‐PR participants were interviewed (mean age (SD) 75 (8) years, 52% male). Four themes were conceptualised: internal motivators influence uptake and adherence, external motivators influence uptake and adherence, programme structure impacts practicality and experience, and living with and managing COPD and other health issues. Motivators for both programmes included improved fitness levels, feeling accountable to the programme and reinforcement from staff and peers. The m‐PR in‐app functionalities such as the daily task list were additionally motivational. m‐PR participants arranged the programme around their schedule while centred‐based participants arranged their schedule around the programme. Multi‐morbidity and illness were barriers to adherence in both programmes. The social network, environment and resources available to participants impacted their enjoyment.

**Conclusion:**

This study adds important information for service providers considering implementation of mHealth PR models.

**Patient or Public Contribution:**

Consumers co‐designed and user‐tested the m‐PR app. The qualitative data presented in this manuscript was obtained through interviews with consumers.

## 1 Introduction

Pulmonary Rehabilitation (PR) is a gold standard intervention for people with chronic obstructive pulmonary disease (COPD) with strong evidence of effectiveness [1]. PR traditionally consists of supervised and individualised exercise training and education, in an outpatient and group setting. Globally, this format, referred to as centre‐based PR (CB‐PR), often has low referral, uptake and completion rates [2, 3, 4].

Enablers of CB‐PR for participants include the improvements in fitness and the motivation received from staff and peers in the group sessions [2, 5, 6]. These enablers are weighed against significant barriers such as the logistical challenges related to set session times and the implications of travelling to a centre such as cost, time and effort [2, 4, 5, 6]. A participant's willingness to complete a programme is influenced by their reality of living with a chronic disease, including fatigue and depression symptoms, managing acute exacerbations and competing commitments, such as attending diagnostic tests and health appointments [2, 4, 5].

To enable better access, PR providers have implemented innovative models of PR such as telerehabilitation models which consist of a remotely delivered rehabilitation service whereby the primary form of contact with a healthcare provider is via virtual care [7, 8, 9]. Virtual care modalities include telephone, videoconferencing, websites or mobile health applications (apps), the latter referred to as mHealth [10]. A systematic review by Cox and colleagues [10] concluded that telerehabilitation models achieve outcomes equivalent to CB‐PR in terms of exercise capacity, health‐related quality of life (HRQoL) and breathlessness. Completion rates for telerehabilitation were higher at 93% compared with 70% in the CB‐PR [10].

Telerehabilitation via mHealth is a model of interest due to the widespread use of smartphones and the comprehensive functions the technology can support [11]. PR participants are willing and able to use smartphone technology to access their rehabilitation [12, 13, 14]. mHealth functions include individualised exercise prescription, in‐built motivational notifications and medication reminders, symptom monitoring, delivery of questionnaires, written or video educational resources, and two‐way communication with health professionals through messaging or audio or video‐conference calls [11, 12, 14].

Internationally, several mHealth PR platforms have been assessed as feasible and acceptable to participants [14, 15, 16, 17, 18, 19, 20, 21]. Cerdan‐de‐Las‐Heras and colleagues [19] compared eight weeks of their mHealth programme with CB‐PR in a randomised controlled trial (RCT). The study demonstrated non‐inferior results between groups for 6‐minute walk distance. Furthermore, high adherence, patient satisfaction and safety were demonstrated at 3‐ and 6‐months follow‐up.

The participant experience has been explored for telerehabilitation models that involved unsupervised exercise sessions with subsequent clinician follow‐up facilitated by telephone [22, 23], videoconference [24] or text messages [21]. One study accompanied this contact with an interactive webpage accessed via a computer tablet [24] and another offered a mHealth app via a smartphone [21]. Similar to CB‐PR, enablers to access included improvements in fitness and social motivation. Telerehabilitation participants spoke positively of including family in their PR experience and finding this, and the contact with staff via virtual means, motivational [21, 22, 24]. An enabler of telerehabilitation was the convenience and flexibility the programme allowed in terms of timing and location of the exercise sessions [21, 22, 24].

In Australia, a team of health and technology experts, together with consumers, have co‐created the first Australian‐specific mHealth PR platform called mobile‐PR (m‐PR). m‐PR, designed as a research prototype, contains a patient app and a clinician web‐based portal. The app underwent user‐testing and sought feedback during design. The majority of participants found m‐PR enjoyable, easy to use and helpful in managing their COPD [14].

Following user‐testing, a RCT was commenced to determine if m‐PR was equivalent to CB‐PR in terms of improvements in exercise capacity, health status and health economics in people with COPD. PR providers must consider the experience alongside the evidence for effectiveness when prescribing these new models of care. To do this, a comprehensive exploration of the participant experience of a mHealth PR programme, and how it compares and differs from CB‐PR is required. Therefore, the aim of this study was to explore the participant experience for people with COPD who undertook either home‐based PR with m‐PR or CB‐PR.

## 2 Methods

This qualitative study involves participants from a large, multi‐centre RCT. This qualitative study and the RCT were approved by the Ethics Committee of the Northern Sydney Local Health District, and the main trial was registered with the Australian and New Zealand Clinical Trial Registry: ACTRN12619001253190. All recruited participants provided written informed consent.

### 2.1 RCT Intervention

The m‐PR and CB‐PR programmes involved an in‐person initial PR assessment and post‐intervention assessment, and 8 weeks of exercise training, disease management education and self‐management support. The interventions are described in detail in the published protocol [25]. The CB‐PR programme involved 2x centre‐based, supervised exercise sessions and one unsupervised home‐based session per week. The m‐PR programme involved 3x unsupervised home‐based sessions per week. The m‐PR app contained a rehabilitation programme of individually prescribed exercises with accompanying videos and the participants had a weekly telephone call with the programme physiotherapist that enabled individualised advice and exercise progression to occur. Additionally, the app provided educational videos covering standard PR topics. In‐app surveys enabled daily and weekly symptom monitoring. Goal setting of behaviours such as step count and a COPD medication action plan were also part of the app.

The m‐PR app relayed data to a clinician web‐based portal where the physiotherapist would monitor the participant's adherence, progress, symptom scores and review the videos that participants had watched. The physiotherapist could update the exercise prescription, change the order or accessibility of videos, update goals and action plans and send personalised in‐app notifications.

### 2.2 Qualitative Study Design

A constructionist epistemology, critical realist ontology and phenomenological methodology were chosen for this qualitative study. Methods included individual semi‐structured interviews and an inductive and reflexive approach to thematic analysis at the semantic level informed by Braun and Clarke [26, 27]. This approach enabled exploration and in‐depth insights into the participant experience of PR, either through m‐PR or CB‐PR [28]. The researchers acknowledged the participants' interaction with their world was informed by the meanings the participants constructed around their experience [29, 30]. Disclosure of the researchers' positionality facilitates reader's understanding of how the authors' experience informed coding and analysis. To this end, nine of the researchers (H. R., M. D., S. W., S. D., S. B., J. A., A. C., I. Y. and Z. M.) had clinical experience in the management of COPD, six authors had PR clinical experience specifically (H. R., M. D., S. W., S. B., J. A. and Z. M.). Seven authors (M. D., S. W., S. D., J. A., A. C., I. Y. and Z. M.) had research experience in COPD and PR, and three authors (M. D., S. D. and R. P.) have qualitative research experience in public health. Influenced by their professional experience, the researchers applied critical analysis and reflection to their analysis, and acknowledged the intervention could be interpreted in different ways by different people [26, 27].

### 2.3 Participants and Recruitment

**Figure 1:**
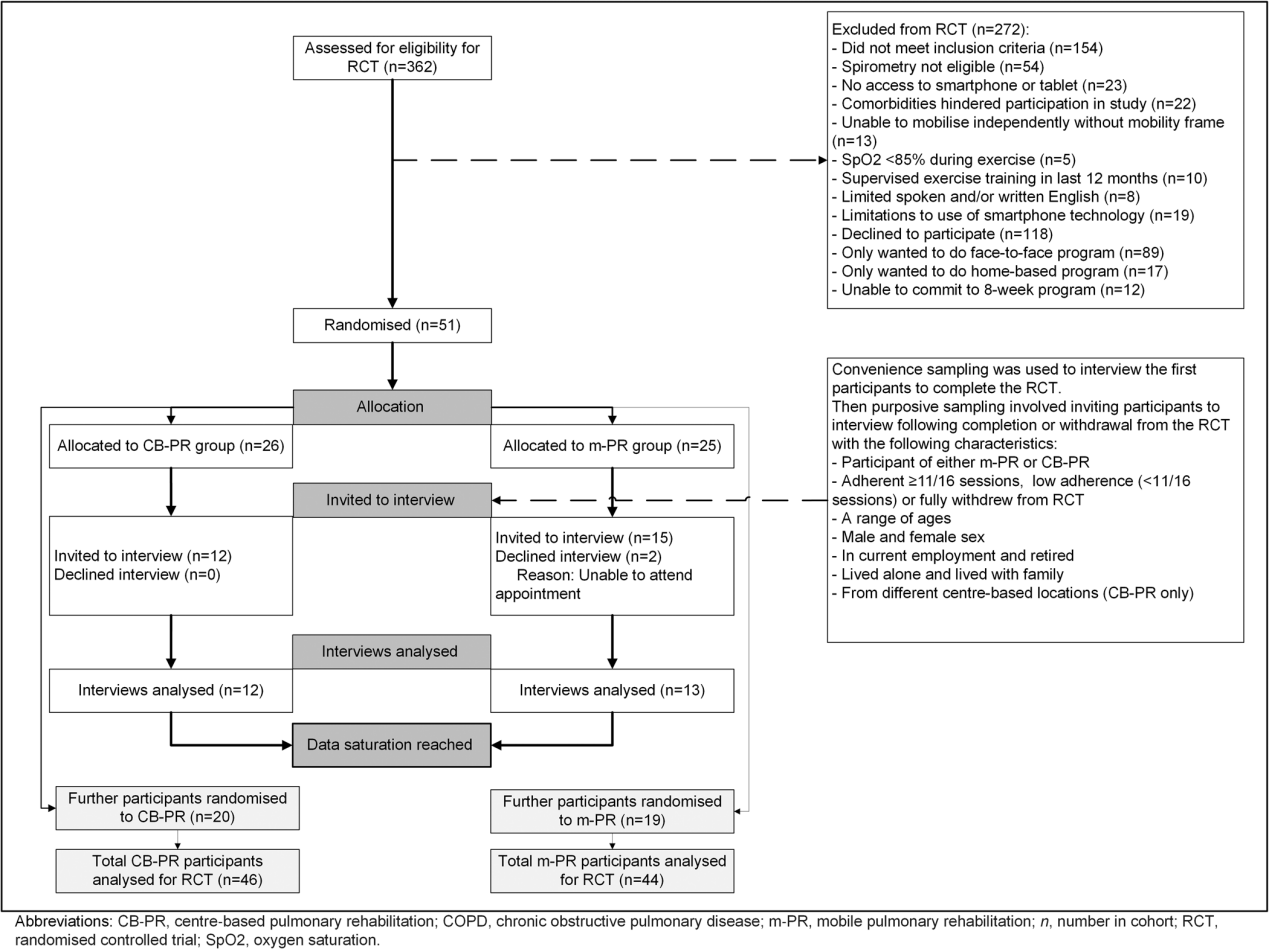
Participant flow and purposive sampling.

The full inclusion and exclusion criteria of the RCT are documented in the published protocol [25]. Convenience then purposive sampling was used to invite participants that completed or withdrew from the RCT to be interviewed. Convenience sampling was used to interview the first four participants to complete the trial. Purposive sampling, detailed in Figure 1, was subsequently implemented to ensure a range of participants with varying characteristics were selected to be interviewed.

Recruitment for this qualitative study occurred between June 2022 and December 2023. Recruitment continued until data saturation was achieved which was identified after 20 interviews (10 m‐PR and 10 CB‐PR). At this stage, four potential themes had been conceptualised from the coded data and answered the research question. A further five interviews were undertaken to confirm that no new codes could be added within the scope of the study. At this time, 51 participants had completed or withdrawn from the RCT and were available to interview.

### 2.4 Data Collection

Participants completed semi‐structured, individual interviews in‐person or via telephone or video conference with the first author [31]. The first author received training to undertake interviews and was not involved in the RCT interventions or known to any participants. An interview guide was developed and internally tested by the research team and field tested with the first participant. Questions were refined to more effectively elicit participants' responses during the internal testing and field‐testing process. The interview guide presented in Table 1, subsequently formed the basis of the following interviews [32]. The interviews were audio‐recorded, de‐identified and transcribed verbatim using a professional transcriber.

**Table 1:** Interview guide.

Introduction: What programme did you take part in?
Q1. How did you feel when you found out which programme you were enrolled into?/Why?
Q2. What were you hoping to achieve when you enrolled in the programme at the start?
Q3. What did you like most about the programme?/Why?
Q4. Were there any parts of the programme you liked less?/Why?
Q5. What did you gain from the programme?
Q6. How satisfied were you with the programme you took part in? Why/why not? Potential probes: Would you recommend it to other people with a similar condition?/what would you say to them?
Q7. Is there anything else about your experience that I have not asked that you would like to add? Potential probes: What feedback do you have about the App itself? Potential probes: Can you tell me about the level of contact and support you had with the staff running the programme?

### 2.5 Data Analysis

Initial familiarisation with, and validation of, the transcribed data occurred by reading the transcripts and listening to the audio recordings [26]. The software package NVivo 14 was used to support the inductive, thematic analysis [33]. For the first ten transcripts, open line by line coding was conducted independently by the first (H. R.) and a second author (M. D.). The authors met regularly to discuss findings and reflections. They grouped these initial codes by identified commonalities in line with a constructivist approach to critical realist ontology. There was strong alignment occurring between their independent coding and any differences were discussed until consensus was attained. The first author open coded the remaining transcripts, aligned codes into potential themes and an evolving thematic map. Reflexivity was undertaken throughout the analysis process, with reflections noted during the coding process and after a session of coding [26]. The thematic map and all coded quotations from the 25 transcripts were subsequently reviewed by the second (M. D.) and a third author (R. P.) to ensure the potential themes adequately reflected the coded data. Through iterative discussions, the three authors reviewed reflective notes and revised the thematic map until final themes were conceptualised. Through critical analysis, they identified definitions and names for the themes and subthemes. This was then discussed with the wider research team.

## 3 Results

**Figure 2:**
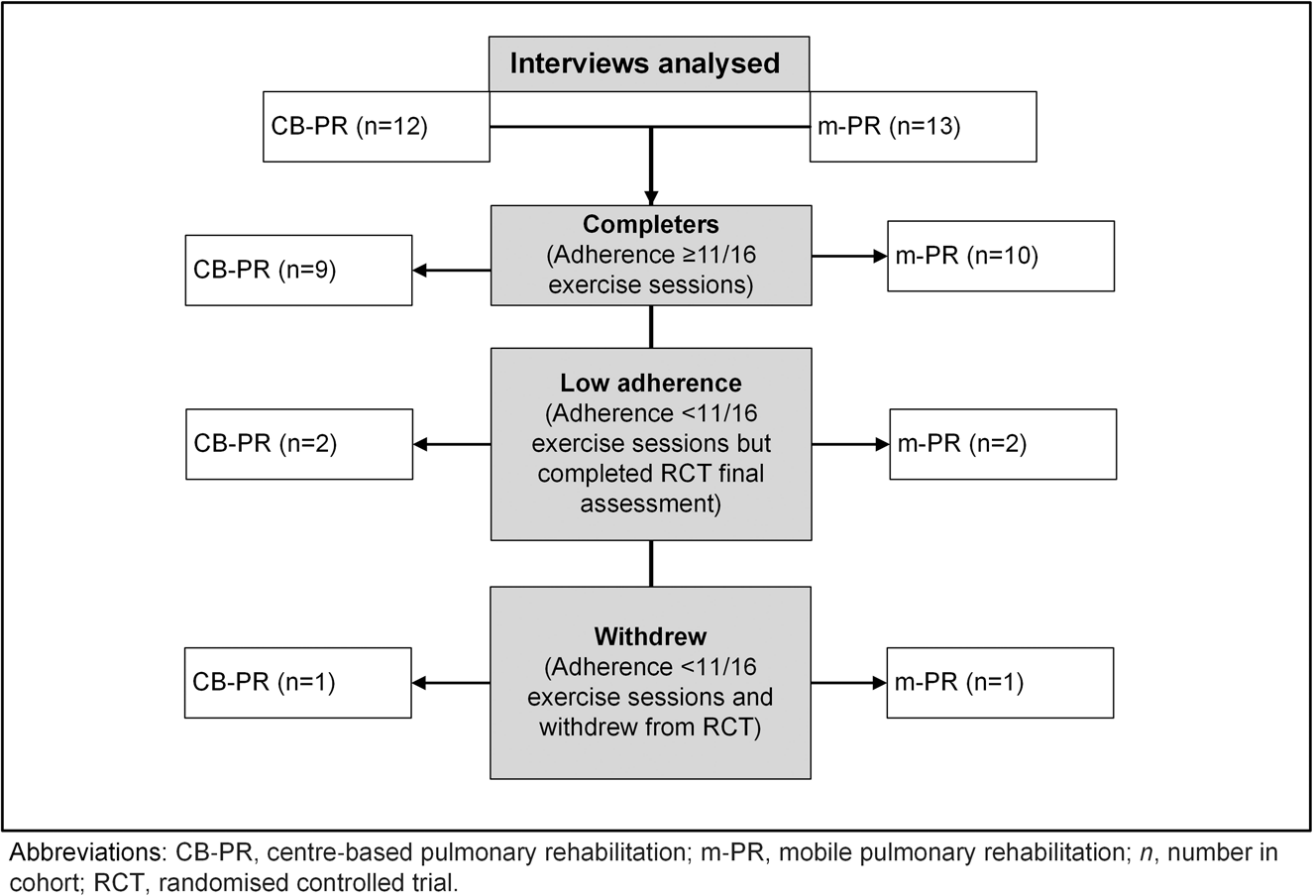
Interviewed participants that completed, had low adherence or withdrew from the RCT.

A total of 27 participants were invited to interview and 25 were interviewed. Of those interviewed, 13 had been randomised to m‐PR and 12 to CB‐PR. For this analysis, participants were considered as ‘completers’ if they completed at least 11 out of 16 centre‐based exercise sessions (70%), and an equivalent number of m‐PR exercise sessions. Figure 2 demonstrates the number of participants that completed the RCT, the number that had low adherence to the exercise sessions but completed the RCT final assessment and the number that withdrew fully from the RCT. The demographics and baseline clinical characteristics of the participants interviewed are listed in Table 2.

**Table 2:** Participants demographics and clinical characteristics.

**Characteristic**		**Combined cohort *n*** = **25**	**m‐PR *n*** = **13**	**Centre *n*** = **12**
Programme completer ≥ 11/16 exercise sessions, *n* (%)	Yes	19 (76)	10 (77)	9 (75)
	No	6 (24)	3 (23)	3 (25)
Age (years), mean (SD)		75 (8)	75 (7)	75 (9)
Sex, *n* (%)	Male	13 (52)	8 (62)	5 (42)
	Female	12 (48)	5 (38)	7 (58)
Employment status *n* (%)	Retired	23 (92)	12 (92)	11 (92)
	Employed	2 (8)	1 (8)	1 (8)
English as preferred language *n* (%)	Yes	24 (96)	12 (92)	12 (100)
	No	1 (4)	1 (8)	0
Lives alone *n* (%)	Yes	10 (40)	4 (31)	6 (50)
	No	15 (60)	9 (69)	6 (50)
Previously participated in PR *n* (%)	Yes	9 (36)	7 (54)	2 (17)
	No	16 (64)	6 (46)	10 (83)
Centre location *n* (%)	Site 1		n/a	6 (50)
	Site 2		n/a	2 (17)
	Site 3		n/a	2 (17)
	Site 4		n/a	2 (17)
Severity of COPD, GOLD category, *n* (%)	1 Mild	2 (8)	1 (8)	1 (8)
	2 Moderate	15 (60)	7 (54)	8 (67)
	3 Severe	7 (28)	5 (38)	2 (17)
	4 Very severe	1 (4)	0	1 (8)
Smoking status	Current smoker	2 (16)	2 (15)	1 (8)
	Ex‐smoker	20 (76)	10 (77)	10 (84)
	Non‐smoker	2 (8)	1 (8)	1 (8)
Presence of co‐morbidities, *n*	Cardiovascular	18	11	7
	Cancer	10	6	4
	Mental	9	5	4
	Metabolic	7	3	4
	Musculoskeletal	16	8	8
	Respiratory	7	4	3
	Other	16	9	7

*Note:* Presence of co‐morbidities: number in cohort with presence of at least one co‐morbidity within category.

Abbreviations: COPD, chronic obstructive pulmonary disease; GOLD, Global Initiative for Chronic Obstructive Lung Disease Criteria; m‐PR, mobile pulmonary rehabilitation; *n*, number in cohort; PR, pulmonary rehabilitation; SD, standard deviation; %, percentage of cohort.

From the data, four themes with associated subthemes were identified across both programmes. All themes were informed by both participants that completed the programmes and participants that had low adherence. The themes were: internal motivators influence uptake and adherence, external motivators influence uptake and adherence, programme structure impacts practicality and experience and living with and managing COPD and other health issues. The themes and subthemes are summarised in Table 3.

**Table 3:** Themes and subthemes associated with experiences of m‐PR and CB‐PR.

1. Internal motivators influence uptake and adherence. Necessity to reclaim and maintain health Feeling a sense of accountability Improvement in fitness and wellbeing Variable levels of enjoyment between programmes
2. External motivators influence uptake and adherence. Integral role of healthcare professional Family and peers promote exercise routine, confidence and competition Goal setting and progress tracking within app (m‐PR only)
3. Programme structure impacts practicality and experience. Flexibility and convenience of programme routine Environment and resources influence experience Familiarisation and use of the app (m‐PR only)
4. Living with and managing COPD and other health issues Acute, chronic and multi‐morbid health issues impact participation Education and self‐management advice appreciated

Abbreviations: CB‐PR, centre‐based pulmonary rehabilitation; COPD, chronic obstructive pulmonary disease; m‐PR, mobile pulmonary rehabilitation.

### 3.1 Theme 1: Internal Motivators Influence Uptake and Adherence

#### 3.1.1 Necessity to Reclaim and Maintain Health

Ten m‐PR and nine CB‐PR participants reported they were motivated to enrol in PR out of feeling a necessity to improve or maintain their current health status—‘*the situation for me was, that [it] was more important for me to do the classes which was a help to me, rather than not*’ #2 (m‐PR, 65 years, male, low adherence). Many acknowledged their health status had deteriorated, and this led to their PR referral. They felt a need for a new option to improve their condition—‘*Because I was feeling really bad, I wanted something to help me, and I was like, I'll try anything*’ #52 (m‐PR, 73 years, female, completed). Some expressed relief to try something other than relying on their medications.

Participants spoke of PR as an important opportunity. They expressed a hope that it would give them some relief in managing their breathlessness and their overall health status.

‘I know that there's not going to be this blinding flash and I'm suddenly going to get better and start running up and down the hills. It's more a matter of living with it and doing what I can to make sure it doesn't get worse’.

#5 (CB‐PR, 74 years, male, completed).

#### 3.1.2 Feeling a Sense of Accountability

Ten m‐PR and nine CB‐PR participants reported feeling accountable to the commitment they made to the programme and personally to the staff. This motivated them to complete the programme—‘*More accountability. Accountability – it's interesting. In my case, it was accountability not only with myself but to the programme itself*’ #16 (m‐PR, 74 years, male, completed).

Participants spoke of the exercise sessions as a personal commitment to which they had chosen to sign up, and they wanted to honour their commitments—‘*If I've made a commitment, I always keep that commitment*’ #4 (CB‐PR, 83 years, female, completed). Participants spoke of a desire to please the staff and do what was expected of them. They referenced how the staff would know if they skipped a session and that knowledge was enough to keep them disciplined in attendance—‘*If I didn't do it for a couple of days, I'd get really cross with myself that I hadn't done it. I was letting [the physiotherapist] down and letting myself down*’ #15 (m‐PR, 78 years, female, completed).

#### 3.1.3 Improvement in Fitness and Wellbeing

Improvements in fitness and wellbeing were reported by seven m‐PR and nine CB‐PR participants. This improvement was noticed early in the programme and motivated the participants to continue—‘*It was just like, oh my gosh, I can't believe how much difference this is starting to make already*’*… and* ‘*I just think that, because I saw how much it benefited me, it's given me that incentive to keep doing it*’ #14 (CB‐PR, 76 years, female, completed).

Participants reported feeling improved breathlessness, energy levels, walking tolerance and some improved their weight—‘*I've got muscles, and not as breathless, and just stronger. I didn't feel so frail*’ #52 (m‐PR, 73 years, female, completed). They noticed an improvement in their mood. They were motivated and confident to participate in more activities. Participants felt proud of their achievements related to their improved fitness and strength—‘*I was worried about taking myself for a walk at first, but after the first couple of walks it was great. [I was] very pleased with myself, sorry but I was. Achieving something, that was good*’ #15 (m‐PR, 78 years, female, completed).

#### 3.1.4 Variable Levels of Enjoyment Between Programmes

Reports of enjoyment experienced during the programme varied between the programmes. Eight m‐PR participants reported that they enjoyed the programme, compared with two from the CB‐PR—‘*[I] loved doing it first thing in the morning. It made you – great way to start the day*’ #16 (m‐PR, 74 years, male, completed). In contrast, seven of the CB‐PR participants spoke of a boredom and lack of enjoyment, compared with two from the m‐PR group—‘*It just made me so irritable in the past when I've had to go on a treadmill. Just incredibly irritable because it's so boring*’ #3 (CB‐PR, 75 years, female, completed).

Enjoyment was linked to participant‐specific preferences and the ability to adjust the sessions to keep them interesting. Preferences for exercising alone or with family or peers were discussed. It was compulsory to exercise with peers in CB‐PR, although, if the participant had access to a social network, the m‐PR programme allowed them to choose to exercise alone or with peers. Other preferences shared were related to the timing of exercise sessions, the environment, resources used and even the music choice during the session. The m‐PR programme enabled flexibility, so more preferences were able to be met and a more interesting and enjoyable experience was had—‘*because we'd go past the golf course. We gradually boosted it up, …try not to do too much, but just push yourself enough basically. It was pleasant*’ #13 (m‐PR, 58 years, male, low adherence). A participant that had previously completed a CB‐PR programme more than a year prior to enrolment in m‐PR commented on his experience of both programmes.

‘The one I did at the hospital I used to go round and round in circles and I thought I was at the Royal Easter Show on the merry‐go‐round. Going round and round and round, round, whereas as you just said, the opposite to—different walking paths and routes and so forth was—I found it quite enjoyable’ … and ‘now when I go by myself, I just put the music in my ears and away I go’.

#11 (m‐PR, 81 years, male, completed)

### 3.2 Theme 2: External Motivators Influence Uptake and Adherence

#### 3.2.1 Integral Role of Healthcare Professionals

Twelve m‐PR and 10 CB‐PR participants emphasised the impact staff had on their uptake and adherence to the programmes. Staff provided education and reassurance at enrolment, they supported them through the initial learning curve of mastering the exercises and, for m‐PR participants, using the app. Participants' motivation was maintained by the staff providing encouragement, addressing concerns quickly and highlighting to participants the improvements that they had made—‘*We talked about did you have a nice weekend? Yes. We just talked about life. And the exercises, of course. Then she motivated me, she was motivating me. Now, you are doing really well and motivating me as well’* #22 (m‐PR, 82 years, female, completed).

m‐PR participants felt supported by the staff. Participants reported it was easy to contact the physiotherapist and the prompt responses were appreciated. The weekly phone calls were keenly anticipated and motivational. CB‐PR participants reported that staff motivated them to increase the intensity at which they exercised within a session—‘*There was no shortage of motivation from the staff when I was there. They were there with a cattle prod*’ #58 (CB‐PR, 76 years, male, low adherence). This was an element noted to be missing by the m‐PR participants that had had prior experience of CB‐PR programmes—‘*Because you're doing it at home, it is more – I think you have to push yourself to be honest. To make sure you do all the exercises as best as you possibly can*’ #13 (m‐PR, 58 years, male, low adherence).

#### 3.2.2 Family and Peers Promote Exercise Routine, Confidence and Competition

Eight m‐PR and nine CB‐PR participants spoke of the motivational influence family and peers had on their experience, and m‐PR participants noted the programme allowed for family involvement. In both programmes, participants' close social circles encouraged them to attend sessions and participants felt proud demonstrating their achievements to them—‘*My eldest works so he's stoked you know, mum, what are you doing, are you at the gym today, what are you doing [unclear] so stoked, yep*’ #24 (CB‐PR, 51 years, female, completed).

For most participants, exercising with peers, either in the centre or with friends or family members at home, promoted confidence, competition and enjoyment within a session—‘*Well, with the girls we catch up, you walk and then you'll have a little talk and a walk. It's a commitment and it's also seeing the girls, you know?*’ #23 (m‐PR, 75 years, female, completed). An m‐PR participant with prior experience of CB‐PR and who exercised alone during the m‐PR programme, acknowledged that she exercised less intensely as a result.

‘If I was in the centre, I know that I'd be being watched. I'm more competitive with other people. An impact for me would be – yeah. I don't know’.

Interviewer: Do you think you push yourself harder in the centre?

‘Yes. I pushed myself much harder’.

#22 (m‐PR, 82 years, female, completed)

#### 3.2.3 Goal Setting and Progress Tracking Within App (m‐PR Only)

In‐app functionalities, such as goal setting, progress tracking and notification reminders, were identified as motivators by 11 m‐PR participants. Recording the activity on the app was rewarding and it reinforced the exercise habit—‘*I just watched the time, and I watched how many steps and thought, no I can do better than that tomorrow. I was in control of that well*’ #15 (m‐PR, 78 years, female, completed).

Many reported they had continued monitoring step count and distance walked after completing the programme. Participants reported a satisfaction in seeing their task list completed at the end of each day—‘*You really wanted to fill everything in, check everything and have it showing up as though you'd done everything. That was quite motivating, the app*’ #52 (m‐PR, 73 years, female, completed).

### 3.3 Theme 3: Programme Structure Impacts Practicality and Experience

#### 3.3.1 Flexibility and Convenience of Programme Routine

Twelve m‐PR participants reported the ability to fit the programme around their routine and the convenience of doing it at home were major benefits of the programme—‘*Because it was on the app, I was able to do it when it was suitable for me and when I felt like doing it*’ #1 (m‐PR, 78 years, male, completed). Participants adjusted the structure and timing of their exercise sessions based on their energy levels and commitments. All the CB‐PR participants spoke of needing to adjust their routine to work around the programme's session times—‘*Well, I just fitted the routine around it. I think probably the—a couple of the things that made it a bit difficult were with made plans*’ #5 (CB‐PR, 74 years, male, completed). For some CB‐PR participants this was not an issue, whereas it became a barrier to completion for others.

The participants that withdrew or had low adherence to the programmes referenced a health issue as their primary reason. However, two of the CB‐PR participants reported the commitment to two classes a week had felt too much for them. One of these participants was employed and struggled to balance the classes with work and the other was a carer for a husband—‘*My husband has got dementia. So, it's pretty difficult, and to get to those places, I now have a [restricted licence], and it was going to also mean involving others to get me there and get me back’* #8 (CB‐PR, 87 years, female, withdrew). In contrast, one of the m‐PR participants was employed with shift work during the programme. Although they missed several sessions due to being unwell, they spoke of how m‐PR allowed them to fit the exercise session in around their changing shift times and when they were feeling well enough.

#### 3.3.2 Environment and Resources Influence Experience

Thirteen m‐PR and nine CB‐PR participants reflected that the quality of the environment and resources available to them impacted their experience. Preferences for exercise environment and equipment varied between individuals. Some preferred fresh air and outdoor spaces and others, controlled, air‐conditioned environments.

‘There's a difference between walking on the flat and knowing that there's not going to be a pothole there or whatever, or tuft of grass, or roll your ankle or whatever. You're a lot more confident when you're walking here than you are at the park. Some of the footpaths are terrible’.

#12 (CB‐PR, 70 years, male, completed)

Preferences for cardiovascular exercise varied between treadmill, stationary bike and walking. For m‐PR participants, the quality of local walking routes, access to larger equipment and exercise space within their homes varied—‘*To make it more interesting, and I'm very fortunate where I live, there's a lot of interesting and good walks round here*’ #16 (m‐PR, 74 years, male, completed). For CB‐PR participants, the newer and more spacious centres supported a more enjoyable experience, but access to equipment could be limited by use by other group members—‘*The trouble with the one at the hospital was it was too many people in too small a space*’ *… and* ‘*I just felt a bit claustrophobic to tell you the truth*’ #50 (CB‐PR, 85 years, male, completed).

#### 3.3.3 Familiarisation and Use of the App (m‐PR Only)

Eleven m‐PR participants reported apprehension about using the app at enrolment. Many had not used an app like this before and were unsure what to expect—‘*I'm not very technically minded, so I was a bit worried about that, but it was very easy to use*’ #13 (m‐PR, 58 years, male, low adherence). There was a specific technology‐related learning process that participants went through to use the app correctly. Reassurance and guidance from staff were required to support this. Staff were required to troubleshoot and address technical issues when the app did not work as required.

‘I hate change, and if something new comes along I'm very apprehensive about it. That's just me. That's just the way I am and that's all walks of life…. I back off a bit, but with this thing, once [the physiotherapist] pointed it out and sat down with me and we had a couple of hiccups early where I couldn't get into it, and (they), but (they) said that wasn't working, it was some problem with the people who set it up, but that was only the first couple of weeks and after that it was all sweet sailing’.

#11 (m‐PR, 81 years, male, completed)

### 3.4 Theme 4: Living With and Managing COPD and Other Health Issues

#### 3.4.1 Acute, Chronic and Multi‐Morbid Health Issues Impact Participation

Twenty‐four participants reported a health issue impacted their participation. Programme schedules were interrupted, or in some instances ceased altogether, due to acute illnesses, exacerbations and hospitalisations related to COPD and other health concerns.

‘It wasn't COVID because I didn't have a fever but gee, I felt bad for–‐ it was a week and a half at least. So, I think I missed three classes, which were then tacked on at the end for me’.

#3 (CB‐PR, 75 years, female, completed)

From the six participants that had low adherence to their programme, five reported this was primarily due to an acute illness and one due to severe osteoarthritis that impacted their ability to participate in the exercises.

‘I got quite sick and then I couldn't do the sessions that were being listed. I was needing time and as time went on, I got sicker and then I ended up in hospital. So really, I think I only got through the first couple of weeks. I didn't get to finish the course’.

#2 (m‐PR 65 years, male, low adherence)

Participants spoke of their complex and multi‐morbid health conditions. They were balancing appointments with multiple specialists. Some were required to have cardiac or other health issues reviewed before they could commence the programme—‘*My blood pressure was too high so they knocked it on the head, and I had to go to the doctors and get a letter from the doctor so that I could continue the programme*’ #6 (CB‐PR, 74 years, male, completed). Throughout the programme, exercise prescriptions were adapted to cater for general aches, pains, and new or chronic injuries—‘*As the walking got longer, because a lot of what was stopping you walking was my peripheral neuropathy that flared up in about the sixth week mark, and that made it a bit harder*’ #1 (m‐PR, 78 years, male, completed).

#### 3.4.2 Education and Self‐Management Advice Appreciated

Ten m‐PR and seven CB‐PR participants spoke of gaining something from the education and self‐management advice they received from the programme staff, and additionally from the videos on the m‐PR app. The specific advice that was most appreciated varied across individuals.

‘I think it was learning. It wasn't just turning up and doing the exercise. It was learning some of the reasons as to why you were doing it. You know, as I said, building up your muscle strength. It hadn't really—I hadn't really related that directly to the ability to breathe or breathe more easily. So, I think it was that interactive component that was really, really beneficial’.

#5 (CB‐PR, 74 years, male, completed)

Some appreciated unscheduled, personal advice from the staff and others found the explanations and videos of the exercises to be important facilitators—‘*Yeah, well, they [the videos] all did something towards helping me breathe, helping me think about anxiety and just how the body works’* #1 (m‐PR, 78 years, male, completed).

## 4 Discussion

This is the first study to comprehensively explore the participant experience of a mHealth PR programme (m‐PR) and provide a comparison with the experience of CB‐PR for people with COPD. Internal and external motivators to participation were similarly present in both programmes, but in‐app functionalities provided unique motivators to the m‐PR participants. Our results demonstrate the m‐PR programme was convenient and flexible. It caused less disruption to an established routine compared to CB‐PR. The programme's flexibility allowed more personal preferences related to programme structure to be met, which led to more enjoyment being reported. Multi‐morbidity and illness were barriers to completion in both programmes.

The first theme revealed that there were variable levels of enjoyment reported by participants in m‐PR and the CB‐PR programmes. The participant responses suggest the social network, quality of the environment and resources available to participants impacts their experience and the enjoyment they report. Enjoyment can be a strong intrinsic motivator for exercise because it provides an immediate reward to participants, can lower the perceived effort levels and support self‐efficacy related to exercise [34]. The enabler of exercising with peers has been reported in prior studies on the participant experience of centre‐based and telerehabilitation programmes [4, 5, 22, 35, 36]. For the m‐PR participants, exercising with other people was only an option if they had access to an appropriate social network from home. Specific elements of an exercise environment have also been linked to positive experiences in prior studies [37]. In a study on a community‐based programme that had views of parklands and a wide range of equipment allowing for variation and preference, participants were noted to be more engaged with the programme [37]. Other studies have shown exercising outdoors and in‐nature improves wellbeing and commitment, reduces symptoms of anxiety and depression and increases engagement with local communities [22, 38]. In the current study, a pleasant and more enjoyable experience was noted by participants attending the newer and more spacious centres or, for m‐PR participants, if they had easy access to walking routes or an appropriate area within their home to exercise.

The role of the physiotherapist was a strong facilitator to adherence identified in Theme 2. In total, 12 m‐PR and 10 CB‐PR participants in our study highlighted how they found the support from staff integral. Staff were noted to facilitate the orientation to the programme and motivate and encourage participants to persevere through to completion. Emerging telerehabilitation models have varying levels of staff contact. In a study by Candy and colleagues [17], the likelihood of completing a programme was nearly twice as high if participants were enrolled in a CB‐PR that transitioned to a telerehabilitation programme supported by telephone and video conferencing compared to a telerehabilitation programme that had no scheduled health care professional contact after enrolment until discharge [17]. The findings of the current study suggest the regular clinician contact in m‐PR, together with the initial face‐to‐face assessment, is sufficient to develop and maintain an effective participant–clinician relationship. This relationship facilitated a feeling of accountability that motivated participants to adhere to the m‐PR programme.

The m‐PR clinician additionally played an integral role in overcoming barriers associated with the familiarisation and use of the app, identified in Theme 3. The study population was from an area with high digital literacy with residents of included suburbs reported to have a digital inclusion index higher than the national average. Despite this, the pre‐RCT user‐experience testing of the m‐PR platform [14] highlighted that challenges with the use of the technology were experienced by some participants and the participants of the current study noted they were apprehensive about using the technology at commencement. Technology related challenges have been reported in other studies of the participant experience of telerehabilitation programmes [21, 23, 24]. Perceived barriers to technology use by people with COPD include a worry about how difficult the technology will be to use [39, 40]. Other barriers include a lack of perceived benefit and a concern that it may replace the opportunity to share information directly with health care professionals [39, 40]. The results of the current study suggest the participants, that were willing to use their smartphones to access their rehabilitation, still appreciated the reassurance, orientation to the technology and assistance with related issues that was offered by staff.

A barrier to completion of CB‐PR, included in Theme 3, was the management of logistics. Transport time, cost and effort and the requirement to prioritise the sessions around prior commitments, such as carer duties or work, are well‐documented challenges for people attending CB‐PR [2, 4, 5, 6]. Participants in this study reported similar experiences. In contrast, a major facilitator identified in the current study was its convenience and ability for participants to fit m‐PR around their schedule. Our study shows how this convenience enabled a flexibility that allowed for individualisation of the session structure and how this also influenced the enjoyment reported. The ability to complete parts of the programme with friends and family and alter the timing and structure of sessions to include different walking routes, and a mix of indoor and outdoor activity, permitted variation which kept the programme interesting.

Lastly, another known barrier to PR completion is the impact of living with a chronic disease [2, 4, 5]. Theme 4 demonstrates that 24 participants interviewed discussed the impact of health issues. People with COPD commonly have co‐morbidities which are becoming more complex with an increased proportion of the population in older age groups. Studies are predicting that both the morbidity and mortality of COPD is rising [41]. Service providers need to predict and manage these issues as they arise throughout a programme. m‐PR participants spoke of how integral the staff contact was to their experience due to the ease in contact and the advice and adjustment to the exercise prescription they provided throughout their programme in response to changes in their physical health. This highlights again the importance of continued therapist involvement whilst delivering digital PR models.

### 4.1 Strengths and Limitations

A strength of this study was the critical realist and constructivist approach which ensured analysis and interpretation of the experience incorporated an appreciation of the participants' demographics and individual contexts. Through this approach, it was evident that themes were impacted by contextual factors such as social living arrangements, employment status, the exercise environment available to participants and their preferences. A limitation to the study may be that baseline lifestyle factors such as nutritional intake and usual involvement in exercise were not assessed for all participants. However, we know the recruited cohort were mostly ex‐smokers with moderate to severe COPD and a range of co‐morbidities (Table 2).

Trustworthiness of our results are demonstrated through our clear description of the methodological process. The authors' experience in PR and qualitative analysis provided the clinical knowledge and skills to appropriately analyse data of populations with COPD. The use of quotes in this manuscript demonstrates how our results are data driven. Credibility is demonstrated through our alignment with previous published literature and the understanding that this study adds to current knowledge.

Limitations to the study include the limited number that had low adherence to the programmes however, these participants highlighted barriers to completion that were consistent with results from prior studies. There were more participants in the m‐PR programme than CB‐PR who had prior PR experience, although none had completed PR within 12 months of their enrolment in the RCT. Their comments gave an interesting insight related to how they felt the intensity to which they exercised could lessen without the direct supervision of a therapist to motivate them. The study may be limited in its transferability to people with similar backgrounds to that specified in the inclusion criteria for the RCT and specifically to people with reasonable digital literacy, access to a smartphone and are willing to use this technology to access their PR. The research was undertaken within one local health district that services a relatively affluent area of metropolitan Sydney, with relatively little cultural diversity in the sample of participants.

## 5 Conclusion

The study adds important information to enhance the implementation of mHealth PR models. The results demonstrate how the convenience of telerehabilitation models may overcome significant barriers of CB‐PR. The impact of multi‐morbidity and acute illness needs to be managed by staff throughout a PR programme. Individual preferences, access to a social network, environment and resources may impact the enjoyment reported and subsequent preferred model choice for participants.

## Author Contributions

**Hannah Rutherford:** conceptualisation, investigation, writing – original draft, methodology, project administration, writing – review and editing, data curation, formal analysis. **Marita Dale:** conceptualisation, methodology, writing – review and editing, formal analysis, data curation, supervision, validation, project administration, investigation, resources, funding acquisition. **Sally Wootton:** conceptualisation, funding acquisition, methodology, validation, writing – review and editing, project administration, supervision, resources. **Rashmi Pithavadian:** conceptualisation, methodology, validation, writing – review and editing, formal analysis. **Sarah Dennis:** methodology, supervision, writing – review and editing, validation. **Sarah Brown:** writing – review and editing, project administration, resources. **Jennifer A. Alison:** writing – review and editing, funding acquisition, resources. **Andrew S. L. Chan:** writing – review and editing, funding acquisition, resources. **Ian A. Yang:** writing – review and editing, funding acquisition, resources. **Zoe McKeough:** conceptualisation, funding acquisition, writing – review and editing, methodology, project administration, supervision, resources, validation.

## Ethics Statement

This research was conducted in compliance with the conditions of the ethics committee approval and the National Health and Medical Research Council, National Statement on Ethical Conduct in Human Research (NHMRC, 2007). The study has been approved by the Northern Sydney Local Health District Human Research Ethics Committee (2019/ETH00368).

## Consent

All participants provided written informed consent.

## Conflicts of Interest

The authors declare no conflicts of interest.

## Data Availability

The data that support the findings of this study are available from the corresponding author, (H.R.), upon reasonable request.
